# Supplementary material for: Computer simulation approach to the identification of visfatin-derived angiogenic peptides
Source: PLoS One. 2023 Jun 29;18(6):e0287577. doi: 10.1371/journal.pone.0287577 (PMC10309634; doi:10.1371/journal.pone.0287577)
Supplement: S1 Table — (DOCX) [file pone.0287577.s001.docx]

Table S1. Toxicity and hemolytic activity predictions of designed peptides in different models using the SVM method

|  | TrEMBL  (SVM Score) | Prediction | Swiss-Prot (SVM-Score) | Prediction | HemoPI-1  (SVM-score) | Prediction |
| --- | --- | --- | --- | --- | --- | --- |
| Peptide-1 | -0.56 | Non-Toxin | -0.70 | Non-Toxin | 0 | Non-hemolytic |
| Peptide-2 | -0.83 | Non-Toxin | -0.93 | Non-Toxin | 0 | Non-hemolytic |
| Peptide-3 | -0.74 | Non-Toxin | -1.14 | Non-Toxin | 0.51 | hemolytic |
| Peptide-4 | -1.64 | Non-Toxin | -1.41 | Non-Toxin | 0.17 | hemolytic |
| Peptide-5 | -0.66 | Non-Toxin | -0.62 | Non-Toxin | 0.18 | hemolytic |
| Peptide-6 | -1.02 | Non-Toxin | -0.78 | Non-Toxin | 0.23 | hemolytic |
| Peptide-7 | -1.27 | Non-Toxin | -1.07 | Non-Toxin | 0 | Non-hemolytic |
| Peptide-8 | -1.43 | Non-Toxin | -0.67 | Non-Toxin | 0.41 | hemolytic |
| Peptide-9 | -0.61 | Non-Toxin | -0.21 | Non-Toxin | 0.02 | hemolytic |
