# Supplementary material for: Computer simulation approach to the identification of visfatin-derived angiogenic peptides
Source: PLoS One. 2023 Jun 29;18(6):e0287577. doi: 10.1371/journal.pone.0287577 (PMC10309634; doi:10.1371/journal.pone.0287577)
Supplement: S2 Table — (DOCX) [file pone.0287577.s002.docx]

Table S2. Toxicity predictions of designed peptides in different models using the QM method

| Peptide | Dipeptide  (Swiss-Prot) | Prediction | Dipeptide  (TrEMBL) | Prediction | Monopeptide  (TrEMBL) | Prediction | Monopeptide  (Swiss-Prot) | Prediction |
| --- | --- | --- | --- | --- | --- | --- | --- | --- |
| -1 | -2.62 | NT | -1.09 | NT | -17.75 | NT | -16.4 | NT |
| -2 | -1.52 | NT | -1.23 | NT | -8.85 | NT | -7.9 | NT |
| -3 | -0.14 | NT | 0.11 | T | -0.69 | NT | -8.8 | NT |
| -4 | -0.63 | NT | -1.20 | NT | -9.43 | NT | -10.9 | NT |
| -5 | 0.93 | T | 0.87 | T | 29.68 | T | 23.4 | T |
| -6 | -0.18 | NT | 0.27 | T | 9.92 | T | 11.7 | T |
| -7 | -0.20 | NT | 0.02 | T | 0.82 | T | 4.6 | T |
| -8 | -0.10 | NT | -2.43 | NT | 1.29 | T | 8.0 | T |
| -9 | 3.32 | T | 3.32 | T | 7.97 | T | 4.1 | T |

Note: T: Toxin, NT: Non-toxin.
