# Supplementary material for: Computer simulation approach to the identification of visfatin-derived angiogenic peptides
Source: PLoS One. 2023 Jun 29;18(6):e0287577. doi: 10.1371/journal.pone.0287577 (PMC10309634; doi:10.1371/journal.pone.0287577)
Supplement: S3 Table — (DOCX) [file pone.0287577.s003.docx]

Table S3: Half-life of Peptides (HLP) in intestine-like environment and blood (Seconds)

|  | HLP  in intestine like environment | Stability | HLP  in Blood |
| --- | --- | --- | --- |
| Peptide-1 | 0.594 | Normal | 805.81 |
| Peptide-2 | 0.594 | Normal | 659.61 |
| Peptide-3 | 0.594 | Normal | 973.71 |
| Peptide-4 | 0.035 | Low | 1280.61 |
| Peptide-5 | 0.035 | Low | 1050.81 |
| Peptide-6 | 0.035 | Low | 921.11 |
| Peptide-7 | 03035 | Low | 975.61 |
| Peptide-8 | 0.742 | Normal | 1335.41 |
| Peptide-9 | 0.594 | Normal | 780.91 |
