# Supplementary material for: Computer simulation approach to the identification of visfatin-derived angiogenic peptides
Source: PLoS One. 2023 Jun 29;18(6):e0287577. doi: 10.1371/journal.pone.0287577 (PMC10309634; doi:10.1371/journal.pone.0287577)
Supplement: S4 Table — (DOCX) [file pone.0287577.s004.docx]

Table S4. Angiogenic and hypertensive activities of designed peptides

|  | SVM Score | AntiAngio-Prediction | SVM Score | AHTpin-prediction |
| --- | --- | --- | --- | --- |
| Peptide-1 | -1.07 | Non-anti-angiogenic | -1.17 | Non-AHT |
| Peptide-2 | -1.01 | Non-anti-angiogenic | -0.30 | Non-AHT |
| Peptide-3 | -0.44 | Non-anti-angiogenic | -0.48 | Non-AHT |
| Peptide-4 | -1.25 | Non-anti-angiogenic | -0.58 | Non-AHT |
| Peptide-5 | -1.00 | Non-anti-angiogenic | -0.17 | Non-AHT |
| Peptide-6 | -0.42 | Non-anti-angiogenic | -0.18 | Non-AHT |
| Peptide-7 | -1.18 | Non-anti-angiogenic | -0.01 | Non-AHT |
| Peptide-8 | -0.05 | Non-anti-angiogenic | -1.03 | Non-AHT |
| Peptide-9 | 0.61 | Anti-angiogenic | -0.25 | Non-AHT |
