# Supplementary figures and images for: Computer simulation approach to the identification of visfatin-derived angiogenic peptides
Source: PLoS One. 2023 Jun 29;18(6):e0287577. doi: 10.1371/journal.pone.0287577 (PMC10309634; doi:10.1371/journal.pone.0287577)

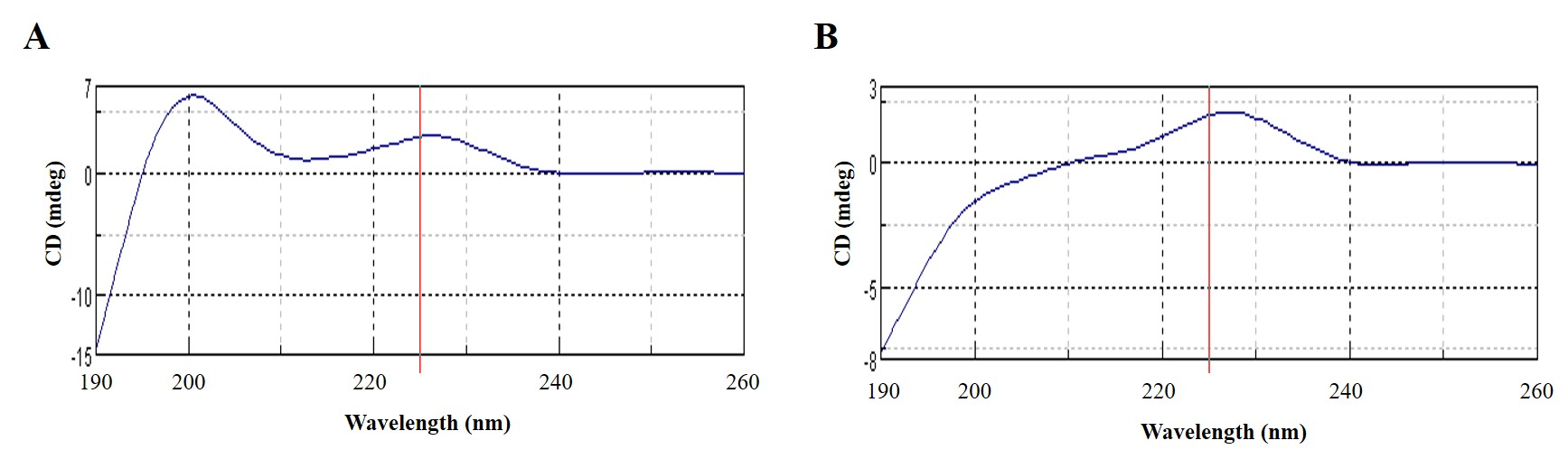

Supplement: S1 Fig — Circular dichroism (CD) spectroscopyof peptide-1 (A) and peptide-2 (B). (TIF) [file pone.0287577.s005.tif]
